# The effect of morning or evening fasted cycling on gastrointestinal function and appetite and metabolic responses in healthy males with overweight

**DOI:** 10.1113/EP092078

**Published:** 2025-03-11

**Authors:** Victoria J. McIver, Lewis R. Mattin, Gethin H. Evans, Adora M. W. Yau

**Affiliations:** ^1^ Department of Life Sciences Manchester Metropolitan University Manchester UK; ^2^ Department of Sport, Exercise and Rehabilitation Northumbria University Newcastle‐Upon‐Tyne UK; ^3^ School of Life Sciences University of Westminster London UK

**Keywords:** appetite, diurnal variation, fasted exercise, gastric emptying, gut hormones, individuals with overweight, metabolic responses

## Abstract

Combining fasting with exercise may influence gastric emptying rate (GER) and provide benefits to weight management and metabolic health. Furthermore, the time of day in which exercise is performed may also influence these variables. The aim was to investigate if fasting or fed exercise at different times of the day would alter GER, appetite and metabolic responses. Twelve males with overweight completed four experimental trials in a randomised crossover fashion involving cycling exercise in the morning fasted (FASTED‐AM), evening fasted (FASTED‐PM) and after a standardised meal in the morning (FED‐AM) and evening (FED‐PM). GER of a semi‐solid meal was measured using the ^13^C‐breath test for 2 h. Appetite hormones, metabolic markers and subjective appetite were measured throughout, with energy intake (EI) monitored for the following 24 h. No difference was observed for GER between trials. No differences were seen between trials for appetite hormone responses except pancreatic polypeptide hormone incremental area under the curve (iAUC) was greater in FED‐PM compared to FASTED‐AM and FASTED‐PM (*P *< 0.05). Glucose concentrations were greater in the postprandial period of FASTED‐PM compared to all trials (*P *< 0.05). No differences in other metabolic marker responses were seen between trials. GER in individuals with overweight was not sensitive to a diurnal variation following fasted or fed exercise, and an acute bout of fasted exercise did not evoke compensatory effects on appetite responses or 24 h EI. Glucose control may be impaired with FASTED‐PM exercise. Future work is required to assess the long‐term impact of fasted exercise on gastrointestinal function, appetite regulation and metabolic health.

## Abstract (highlights)

- **What is the central question of this study?**
  Does fasted exercise at different times of the day affect gastric emptying rate, appetite and metabolic responses compared to fed exercise in individuals with overweight?
- **What is the main finding and its importance?**
  The time of fasted exercise did not result in differences in gastric emptying rate compared to fed exercise. However, evening fasted exercise may lead to poorer glucose control during a subsequent meal. Energy intake was not increased in the 24 h following in compensation, therefore, combining fasting with moderate‐intensity exercise may be a potential strategy for weight management practices.

## 1 INTRODUCTION

With the increasing prevalence of individuals with overweight or obesity reaching epidemic levels worldwide, simple interventions are needed to manage and/or prevent this and alleviate the impact on public health and healthcare systems (Koliaki et al., 2023). Intermittent fasting, which involves alternating between periods of eating and fasting or extending periods of time without eating as an approach to restricting calorie intake, has been shown to be an effective strategy for weight loss (Patikorn et al., 2021; Welton et al., 2020). Exercise in the fasted state may be one such intervention, as the combination of intermittent fasting and exercise affects both energy intake (EI) and energy expenditure (EE) simultaneously (Aird et al., 2018). Previous work investigating the effect of fasted exercise on appetite regulation has found minimal changes in appetite hormone responses with no differences observed in peptide tyrosine tyrosine (PYY), glucagon‐like peptide‐1 (GLP‐1) or acylated ghrelin responses post‐subsequent meal ingestion following fasted exercise compared to fed exercise (McIver et al., 2019a, 2019b). However, these previous studies were conducted on individuals with a mixture of body compositions, which could be a potential reason for the small or no differences observed. Body composition may alter subjective appetite, appetite‐regulatory hormones and EI following exercise. Studies have shown greater adiposity is related to higher fasting levels of acylated ghrelin and lower levels of PYY and GLP‐1, whilst postprandial changes are less sensitive (Coutinho et al., 2018; Karra & Batterham, 2010). Furthermore, leptin and insulin concentrations have been shown to be elevated with greater adiposity (Considine et al., 1996; Coutinho et al., 2018; Karra & Batterham, 2010; Bagdade et al., 1967). On the other hand, Douglas et al. (2017) found subjective appetite, appetite hormone responses and *ad libitum* EI to be similar between lean individuals and individuals with overweight/obesity. Therefore, a need exists to explore how fasted exercise influences appetite regulatory variables within a specific adiposity‐targeted cohort.

Increasing evidence suggests that circadian rhythms, nutrition and metabolism are intimately linked. Circadian rhythms regulate much of gastrointestinal physiology, such as motility, digestion and absorption (Voigt et al. 2019). Some evidence suggests that gastric emptying rate (GER) follows a diurnal pattern, with delayed gastric emptying in the evening versus morning (Goo et al., 1987; Grammaticos et al., 2015). Some of our previous work is also consistent with this, which found that evening fasted exercise results in delayed GER, with no changes in appetite (McIver et al., 2019a). However, the existing studies that have explored GER at different times of day have used either healthy average weight or individuals with mixed adiposity (McIver et al., 2019a; Goo et al., 1987; Grammaticos et al., 2015). Whether individuals with overweight are also sensitive to this diurnal variation pattern previously observed in GER is unknown. Therefore, it would be advantageous to explore whether GER follows a diurnal variation following fasted exercise and whether there is an optimal time for performance of exercise in the fasted state on appetite regulation and GER in individuals with overweight.

Therefore, the aims of this study were to (1) investigate the effects of fasted exercise versus non‐fasted exercise on GER, appetite regulatory hormones and metabolic responses in males with overweight and (2) explore the diurnal variation of these outcomes in response to fasted exercise in individuals with overweight. It was hypothesised that there would be differences in GER, appetite hormone responses and metabolic responses between fasted versus non‐fasted exercise, which may be modulated by the different times of the day.

## 2 METHODS

### 2.1 Ethical approval

All subjects were informed of the details of the study procedures, both verbally and in writing, before providing their written informed consent. All procedures followed were conducted according to the *Declaration of Helsinki* (version 2013) and were granted approval by the Faculty of Science and Engineering Research Ethics and Governance Committee of Manchester Metropolitan University before commencement (Reference number: 0878). This study was publicly registered with the ISRCTN database (ISRCTN11880065; https://www.isrctn.com/ISRCTN11880065).

### 2.2 Participants

Twelve males with overweight volunteered to participate in this study. Individuals were classified as overweight according to satisfying at least one of the conditions of BMI between 25.0 and 29.9 kg/m^2^ (according to WHO, 2010) or a body fat percentage between 18% and 27% (Barba et al., 2004) in cases where BMI was marginally greater than the overweight cut off. A sample size of 12 was determined to be adequate based on power analyses using data that would result in a detectable change in GER (McIver et al., 2019a; Goo et al., 1987), fat oxidation (Iwayama et al., 2015) and total EI (Bachman et al., 2016) with 80% power and at a significance level of 5%.

Participant inclusion criteria comprised of male individuals aged 18–40 years old, not taking regular medication and having no known history of respiratory, cardiovascular, endocrine or chronic gastrointestinal disorders or disease as assessed by a medical screening questionnaire, classified as moderate or intermediate chronotypes (i.e., not extreme chronotypes) according to the Munich chronotype questionnaire (Roenneberg et al., 2003), free from musculoskeletal injury, non‐smokers and were not dieting. In addition, participants were not involved in shift work and did not report any disturbances to their normal sleep–wake cycle during the 1 week before data collection. Participant characteristics are shown in Table 1.

**TABLE 1:** Participant characteristics (*n* = 12 participants).

Characteristic	Value
Age (years)	26 ± 4
Height (cm)	177.9 ± 7.7
Weight (kg)	88.58 ± 11.20
BMI (kg/m^2^)	28.0 ± 2.6
Body fat (kg)	23.9 ± 4.2
Fat free mass (kg)	64.3 ± 8.3
Body fat (%)	23.2 ± 2.4
Fat free mass (%)	77.1 ± 1.8
${\dot{V}}_{O_{2}\mathrm{peak}}$ (ml/kg/min)	42.3 ± 6.0
Self‐reported PA levels per week (min)	168 ± 19
Systolic BP (mmHg)	128 ± 8
Diastolic BP (mmHg)	87 ± 6

*Note*: Values expressed as mean ± SD. Abbreviations: BMI, body mass index; BP, blood pressure; PA, physical activity.

### 2.3 Preliminary trial

All participants attended a preliminary trial with no requirement to fast at least 7 days before the first experimental trial. Anthropometric measures of height, body mass and body fat percentage, as well as familiarisation with the breath sampling procedures, were completed. Height was measured to the nearest 0.1 cm using a wall‐mounted stadiometer, and body mass to the nearest 0.01 kg using electronic scales (GFK 150; Adam Equipment Co. Ltd, Milton Keynes, UK). Body fat percentage was measured using bioelectrical impedance analysis (Omron BF306; Kyoto, Japan). During this visit, participants also completed a physical activity and dietary habit questionnaire, the Munich Chronotype Questionnaire (Roenneberg et al., 2003), the Pittsburgh Sleep Quality Index (Buysse et al., 1989) and the Epworth Sleepiness Scale (Johns, 1991).

Participants also completed a ${\dot{V}}_{O_{2}\mathrm{peak}}$ test on a cycle ergometer (Corival CPET, Lode, The Netherlands). The protocol initially commenced with a 5 min warm‐up with workload set on 0 W, followed by increments of 1 W every 2 s until volitional exhaustion. Expired air was continuously analysed using a breath‐by‐breath gas analyser (Oxycon Pro, CareFusion, Leipzig, Germany). Respiratory gas exchange measures were averaged every 30 s with ${\dot{V}}_{O_{2}\mathrm{peak}}$ determined as the highest of the two 30‐s periods within the final 1‐min period of the exercise session. Heart rate was measured continuously using a heart rate monitor (Polar H7, Kempele, Finland) and rating of perceived exertion (RPE) (Borg, 1982) was recorded every 3 min. Before leaving the laboratory, all participants were provided with food weighing scales and asked to record their physical activity and food intake in the 24 h before the start of their first experimental trial. They were then asked to replicate their activity and diet the day preceding their subsequent trials. In addition, they were asked to refrain from alcohol consumption, strenuous exercise and caffeine ingestion 24 h before trials. Participants were also asked to complete a 7‐day sleep diary leading up to each trial. The midpoint of sleep was calculated by identifying the clock time midway between the time participants fell asleep and their waking time.

### 2.4 Experimental trials

All participants completed four experimental trials in a randomised crossover fashion: two morning trials (08.00 –13.00 h) fasted (FASTED‐AM) and non‐fasted (FED‐AM) and two evening trials (15.00–20.00 h) fasted (FASTED‐PM) and non‐fasted (FED‐PM). Trials were separated by a minimum of 7 days.

In the morning trials, the participants were required to fast from midnight, and in the evening trials they were required to have their usual breakfast and then fast from 07.00 h, except for plain water consumption. Baseline measurements of body mass, blood samples, appetite, substrate utilisation and saliva samples were made and collected. Participants were asked to empty their bladder before the measurement of body mass to the nearest 0.01 kg using electronic scales (GFK 150; Adam Equipment Co. Ltd). Appetite was assessed using 100 mm visual analogue scales (VAS) (Flint et al., 2000). Ratings of hunger, fullness and prospective food consumption (PFC), as well as ratings of food satisfaction, were collected. Substrate utilisation was measured through indirect calorimetry using a breath‐by‐breath gas analyser (Oxycon Pro, CareFusion) with expired air collected for 10 min and the last 5 min of each 10 min segment was used to calculate substrate utilisation using stoichiometric equations (Peronnet & Massicotte, 1991). A saliva sample was collected to measure melatonin concentrations as a circadian phase marker to signify participants were at different stages of their daily rhythms between morning and evening trials.

Following baseline measurements, participants ingested a standardised test meal (test meal 1) in the FED trials within a 15 min period or remained fasted in the FASTED trials. Test meal 1 was composed of typical breakfast components and consisted of 30 g of breakfast cereal (Kellogg's Original Special K) with 125 mL of British semi‐skimmed milk and a croissant (Sainsbury's, UK), providing 351 kcal, 51 g carbohydrate, 11 g fat and 11 g protein. Ratings of appetite and substrate utilisation were measured at the end of the 15 min test meal 1 period. The participants then rested for 1 h before the commencement of the exercise protocol. During this 1 h rest period, further measures of appetite were taken every 15 min and substrate utilisation every 30 min. The exercise protocol involved 60 min of cycling on a Lode Corival ergometer at a workload intensity of 60% ${\dot{V}}_{O_{2}\mathrm{peak}}$ pre‐determined in the preliminary trial (average workload of 139 ± 11 W). Heart rate and RPE were measured every 15 min throughout the exercise, with expired air measured continuously with the last 5 min of each 10 min segment used to calculate substrate utilisation. After completion of the exercise bout, the participants recovered for 30 min (showered if desired) before they ingested test meal 2. Test meal 2 was comprised of a typical lunch component, a semi‐solid meal consisting of 800 g of Heinz vegetable soup (1584 kJ (376 kcal), containing 6.8 g fat, 66.4 g carbohydrate, 8.8 g protein) which was ingested within a 15 min period and used to assess GER. Subjective feelings of appetite and substrate utilisation were measured every 15 min post‐ingestion for a total period of 2 h. Twenty‐four hours EI post‐trial was recorded via a weighed‐food diary by all the participants and later analysed using Nutritics Dietary Analysis (Nutritics, Dublin, Ireland). The recording period started immediately from the moment they left the laboratory.

**FIGURE 1:**
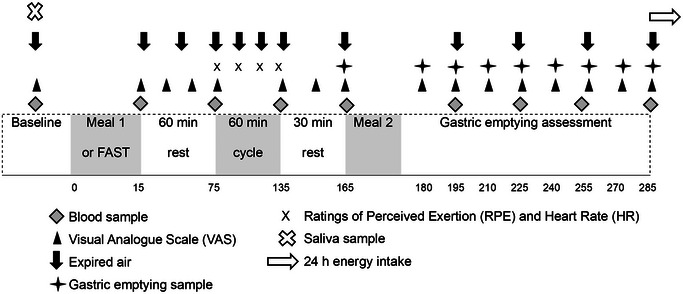
Schematic diagram of the experimental trial protocol, represented in minutes of the trial.

The primary outcome measures were GER and appetite regulatory hormones, metabolic markers, subjective appetite, 24‐h EI and substrate utilisation. Secondary outcome measures were RPE and HR during exercise and salivary melatonin concentrations. A schematic diagram of the experimental protocol is presented in Figure 1.

### 2.5 Blood sampling and analysis

An intravenous cannula (BD Venflon, 20G, Becton Dickinson Infusion Therapy, Helsingborg, Sweden) was inserted into an antecubital vein which remained in place for the duration of the trial. The cannula was kept patent with the infusion of isotonic saline after each sample collection. Blood samples were collected at baseline, post meal 1 period, pre‐exercise, immediately post‐exercise, pre‐meal 2 ingestion, then every 30 min post‐meal 2 for 2 h. To prevent the degradation of acylated ghrelin and active GLP‐1, 50 µL of Pefabloc (Roche Diagnostics Limited, Burgess Hill, UK) and 50 µL of DPP‐IV (Merck Millipore Ltd, Feltham, UK) was immediately added to blood samples, which were kept on ice until all samples were collected. Blood samples were centrifuged at 1500 *g* for 15 min at 4°C and the serum aliquoted and stored at −80°C until analysis. Serum glucose, non‐esterified fatty acids (NEFA), triglycerides and total cholesterol concentrations were determined in duplicate using a clinical chemistry analyser (Randox Daytona, Crumlin, UK). Circulating concentrations of acylated ghrelin, active GLP‐1 (both GLP‐1^7–36^ and GLP‐1^7–37^), total PYY, pancreatic polypeptide (PP) and insulin were determined in duplicate using multiplex analysis (Luminex 200, Luminex Corp., Austin, TX, USA) with kits purchased from Merck‐Millipore (HMHMAG‐34K, Milliplex MAP). Intra‐assay coefficients of variation were: ghrelin, 4.9%; GLP‐1, 8.9%; insulin, 7.0%; PP, 8.2%; PYY, 7.4%; glucose, 1%; triglycerides, 1.1%; NEFA, 4.2%; and cholesterol, 1.1%.

### 2.6 Gastric emptying measurement and analysis

The semi‐solid soup meal contained 100 mg of ^13^C‐sodium acetate for the assessment of GER using the breath test method. An end‐expiratory breath sample was collected pre‐meal ingestion and then at 15 min intervals post‐meal ingestion for 2 h. On each occasion, breath samples were collected into a 100 mL foil bag by exhalation through a one‐way valve mouthpiece (Wagner Analyzen‐Technik, Bremen, Germany). Breath samples were analysed for the ratio of ^13^CO_2_:^12^CO_2_ by non‐dispersive infrared spectroscopy (IRIS Dynamic, Kibion, Germany). The difference in the ratio of ^13^CO_2_:^12^CO_2_ from baseline breath to post‐ingestion breath samples is expressed as delta over baseline (DOB). Half‐emptying time (*T*_½_) and time of maximum emptying rate (*T*_lag_) were calculated utilising the manufacturer's integrated software evaluation incorporating equations of a previously described formula (Ghoos et al., 1993).

### 2.7 Saliva sample and analysis

A saliva sample was collected at the beginning of all trials (AM trials 08.00 h; PM trials 15.00 h) by the passive drool method through a collection aid (Salimetrics Europe Ltd, Newmarket, UK) into the collection tube. Saliva samples were immediately stored at −80°C until analysis. On the day of analysis, saliva samples were thawed, vortexed and then centrifuged at 1500 *g* for 15 min at 4°C. Melatonin concentrations were determined in duplicate using ELISA (Salimetrics, State College, PA, USA).

### 2.8 Statistical analysis

The incremental area under the curve (iAUC) was calculated using the Time Series Response Analyser (TSRA) (Narang et al., 2020). Two‐way repeated‐measures ANOVA was used to assess the presence of trial (fasted vs. fed) × time of day (morning vs. evening) differences in iAUC for gastric emptying DOB, blood serum concentration of hormones and metabolic markers, substrate oxidation and VAS ratings. A two‐way repeated‐measures ANOVA was also used to assess trial (fasted vs. fed) × time of day (morning vs. evening) differences for gastric emptying *T*_½_ and *T*_lag_ data, salivary melatonin concentration and 24‐h EI. A three‐way (trial × time of day × time across trial) repeated‐measures ANOVA was used to assess trial (fasted vs. fed) × time of trial (morning vs. evening) × time across trial differences for gastric emptying DOB, blood serum concentrations of hormones, metabolic markers, substrate oxidation and VAS ratings. Sphericity for repeated measures was assessed, and where appropriate, Greenhouse–Geisser corrections were applied for epsilon <0.75 and the Huynh–Feldt correction was adopted for less severe asphericity. Significant *F*‐tests were followed by one‐way repeated measures ANOVA and/or Bonferroni‐adjusted pairwise comparisons as appropriate. One‐way repeated‐measures ANOVA was also conducted for comparison of body mass between all trials and mid‐point of sleep. All analyses were carried out using IBM SPSS Statistics (v25.0 for Windows; IBM Corp., Armonk, NY, USA). The level of significance was set at *P *< 0.05. Descriptive data are presented as means ± SD.

## 3 RESULTS

### 3.1 GER

**FIGURE 2:**
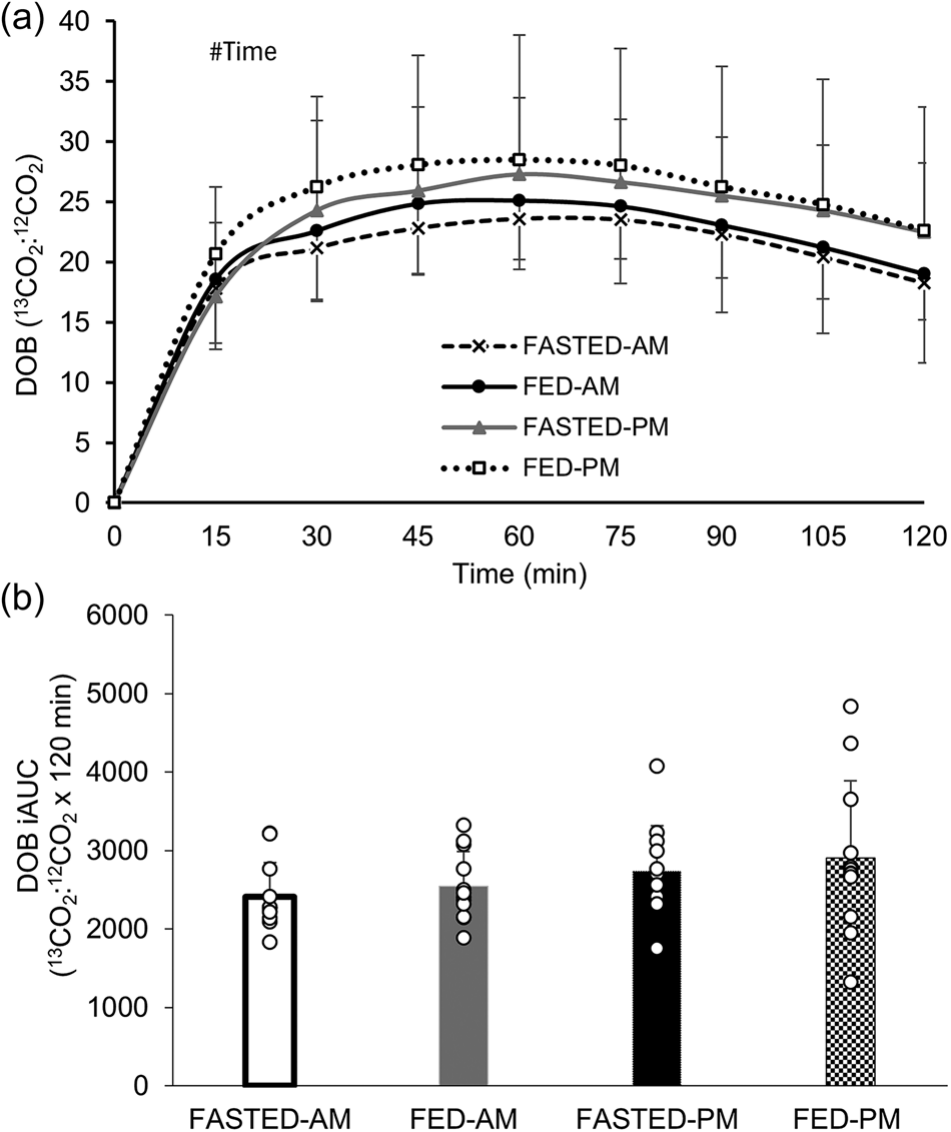
Gastric emptying DOB (a) and gastric emptying DOB iAUC (b) of the semi‐solid test meal 2 (800 g vegetable soup) for all four trials following 60 min of cycling performed either fasted (FASTED) or after test meal 1 consumption (FED) in the morning (AM) or evening (PM). Dashed line (crosses), FASTED‐AM; black continuous line (filled circles), FED‐AM; grey continuous line (filled triangles), FASTED‐PM; dotted line (open squares), FED‐PM. #Main effect of time (*P *< 0.001). Values are means ± SD; *n* = 12 participants. DOB, delta over baseline; iAUC, incremental area under the curve.

**FIGURE 3:**
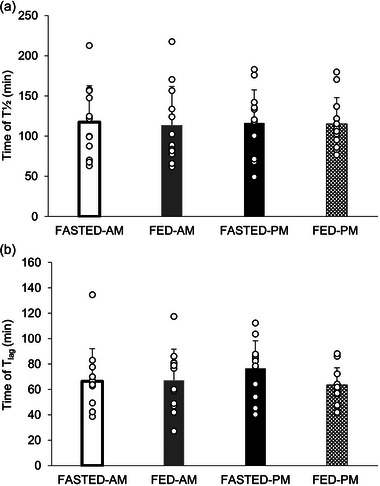
GER *T*_½_ (a) and *T*_lag_ (b) of the semi‐solid test meal 2 (800 g vegetable soup) for all four trials following 60 min of cycling performed either fasted (FASTED) or after test meal 1 consumption (FED) in the morning (AM) or evening (PM). Values are means ± SD; *n* = 12 participants. GER, gastric emptying rate; *T*_½,_ half time; *T*_lag_, time of maximal emptying rate.

No main effect of trial (*P* = 0.410) or time of day (*P* = 0.102) or interaction effect (*P* = 0.705) was observed for DOB (Figure 2a). A main effect of time (*P *< 0.001) was seen, however, with increases in ^13^CO_2_:^12^CO_2_ from baseline, peaking at 60 min before decreasing at the end of the trial. No main effect of trial (*P* = 0.405) or time of day (*P* = 0.109) or trial × time of day interaction effect (*P* = 0.917) was observed for iAUC DOB (Figure 2b). Furthermore, for *T*_½_ (Figure 3a) and *T*_lag_ (Figure 3b), no main effect of trial (*P* = 0.835 and *P* = 0.335, respectively) or time of day (*P* = 0.978 and *P* = 0.556, respectively) or trial × time of day interaction effect (*P* = 0.868 and *P* = 0.133, respectively) was observed.

### 3.2 Gut hormones

**FIGURE 4:**
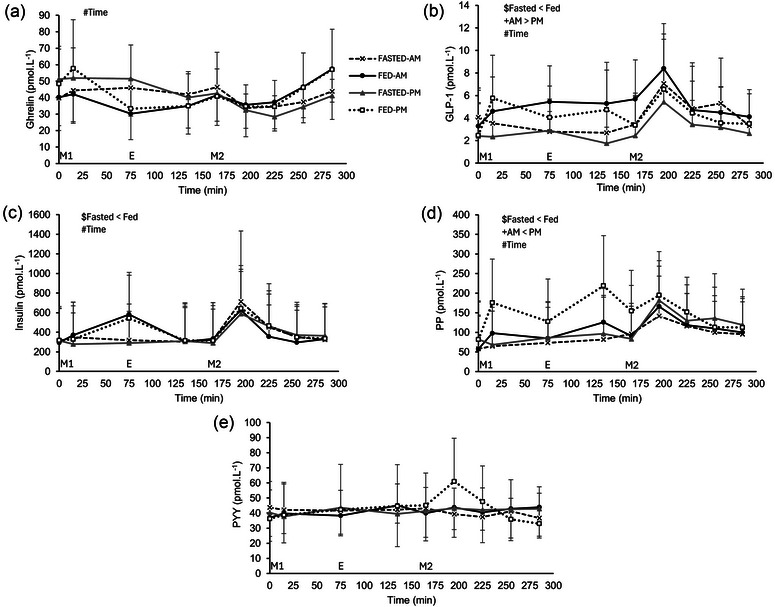
Gastrointestinal hormone responses. Serum concentrations over time of ghrelin (*n *= 12 participants) (a), GLP‐1 (*n *= 11 participants) (b), insulin (*n* = 12 participants) (c), PP (*n =* 11 participants) (d) and PYY (*n* = 6 participants) (e). Trials involved exercise performed in the fasted state (FASTED) or after consumption of test meal 1 (FED) and in the morning (AM) or evening (PM). Dashed line (crosses), FASTED‐AM; black continuous line (filled circles), FED‐AM; grey continuous line (filled triangles), FASTED‐PM; dotted line (open squares), FED‐PM. ‘M1’ indicates ingestion of test meal 1 during the FED trials or continuation of fasting during the FASTED trials; ‘E’ indicates the start of the exercise period, where participants completed 1 h of cycling at 60% of their ${\dot{V}}_{O_{2}\mathrm{peak}}$; and ‘M2’ indicates ingestion of test meal 2 where 800 g vegetable soup was ingested in all trials. Values represent means ± SD. The main effects indicated in the figures: $main effect of trial, +main effect of time of day, #main effect of time (*P *< 0.05). GLP‐1, glucagon‐like peptide‐1; PP, pancreatic polypeptide; PYY, peptide tyrosine tyrosine.

For concentration time course data, no main effect of trial (*P* = 0.628) or time of day (*P* = 0.281) or three‐way interaction effect (*P* = 0.644) was observed for ghrelin concentrations (Figure 4a), but a main effect of time was observed (*P* = 0.001). Ghrelin decreased following ingestion of test meal 1 and increased in the second hour post‐test meal 2 ingestion. Main effects of trial (*P* = 0.004), time of day (*P* = 0.045) and time (*P* < 0.001) were found for GLP‐1 concentrations. However, no three‐way interaction effect was observed (*P* = 0.432). GLP‐1 concentrations were higher in the morning trials compared to the evening (4.6 ± 3.3 vs. 3.6 ± 3.2 pmol L^−1^), lower in the fasted compared to fed trials (3.5 ± 3.0 vs. 4.7 ± 3.4 pmol L^−1^) and higher at 195 min than the majority of time points (*n *= 11 participants, Figure 4b). A main effect of trial (*P* = 0.034) and time (*P* = 0.004), but not time of day (*P* = 0.879) or three‐way interaction effect (*P* = 0.639), was observed for insulin concentrations (Figure 4c). Insulin concentrations were lower in the fasted compared to the fed trials (373 ± 382 vs. 395 ± 376 pmol L^−1^). A main effect of trial (*P* = 0.001), time of day (*P* = 0.002) and time (*P* < 0.001), but no three‐way interaction effect (*P* = 0.124), was observed for PP concentrations. PP concentrations were lower in the morning compared to the evening (99 ± 93 vs. 129 ± 103 pmol L^−1^), lower in the fasted compared to the fed trials (101 ± 98 vs. 127 ± 100 pmol L^−1^) and greater at 225 min compared to 285 min (129 ± 84 vs. 107 ± 83 pmol L^−1^; *P* = 0.028) (*n *= 11 participants, Figure 4d). No main effect of trial (*P* = 0.709), time of day (*P* = 0.830) time (*P* = 0.169) or three‐way interaction effect (*P* = 0.337) was found for PYY concentrations (*n *= 6 participants, Figure 4e).

**FIGURE 5:**
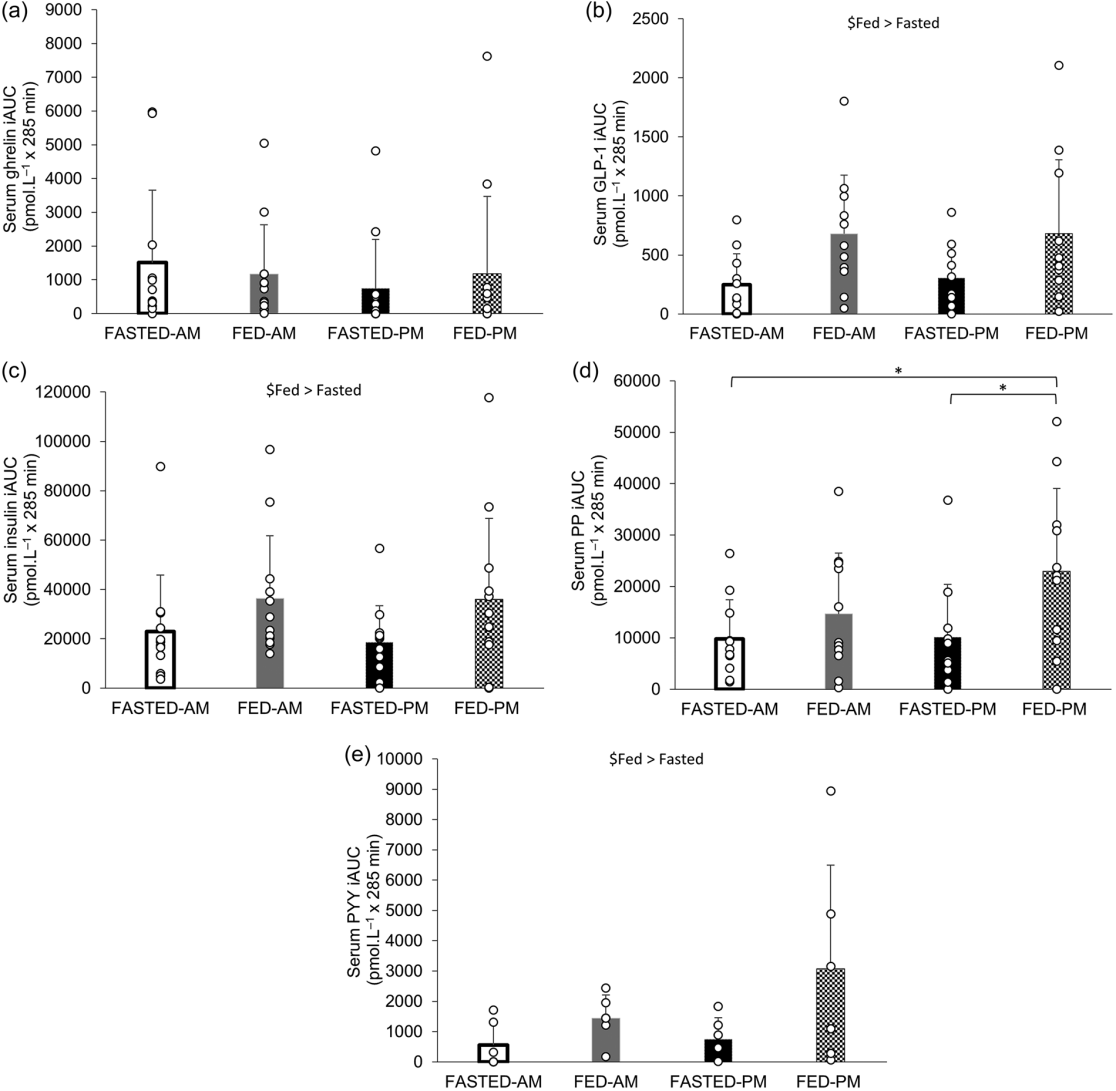
Gastrointestinal hormone responses. iAUC serum concentrations of ghrelin (*n* = 12 participants) (a), GLP‐1 (*n *= 11 participants) (b), insulin (*n *= 12 participants) (c), PP (*n *= 11 participants) (d) and PYY (*n = *6 participants) (e). Trials involved exercise performed in the fasted state (FASTED) or after consumption of test meal 1 (FED) and in the morning (AM) or evening (PM). Values represent means ± SD. $main effect of trial with fed greater than fasted (*P *< 0.05). *Difference between trials (*P *< 0.05). iAUC, incremental area under the curve; GLP‐1, glucagon‐like peptide‐1; PP, pancreatic polypeptide; PYY, peptide tyrosine tyrosine.

No significant effect of trial (*P* = 0.934) or time of day (*P* = 0.314) or trial × time of day interaction effect (*P* = 0.121) was observed for ghrelin iAUC concentrations (Figure 5a). GLP‐1 iAUC concentrations (*n *= 11 participants) showed a main trial effect, with it being greater in the fed trials compared to the fasted trials (680 ± 551 vs. 276 ± 259 pmol L^−1^ × 285 min; *P* = 0.002). No time of day (*P* = 0.814) or interaction effect (*P* = 0.856) was observed, however (Figure 5b). Similarly, a main trial effect (*P* = 0.004), but no time of day effect (*P* = 0.659) or interaction effect (*P* = 0.652), was observed for insulin iAUC. Insulin iAUC concentrations were higher in the fed trials in comparison to the fasted trials (36,146 ± 28,625 vs. 20,797 ± 18,967 pmol L^−1^ × 285 min) (Figure 5c). A main effect of trial (*P* = 0.006) and time of day (*P* = 0.034) and an interaction effect (*P* = 0.021) for serum PP iAUC concentrations was observed (*n *= 11 participants; Figure 5d). PP iAUC concentrations were greater in the FED‐PM trial compared to FASTED‐AM (22,958 ± 16,110 vs. 9770 ± 7640 pmol L^−1^ × 285 min; *P* = 0.033) and FASTED‐PM (vs. 10,108 ± 10,286 pmol L^−1^ × 285 min; *P* = 0.015). For PYY (*n *= 6 participants, Figure 5e), a main effect of trial was observed, with it being higher in the fed trials compared to the fasted (2255 ± 2512 vs. 651 ± 708 pmol L^−1^ × 285 min; *P* = 0.036), but there was no time of day effect (*P* = 0.335) or trial × time of day interaction effect (*P* = 0.390).

### 3.3 Metabolic markers

**FIGURE 6:**
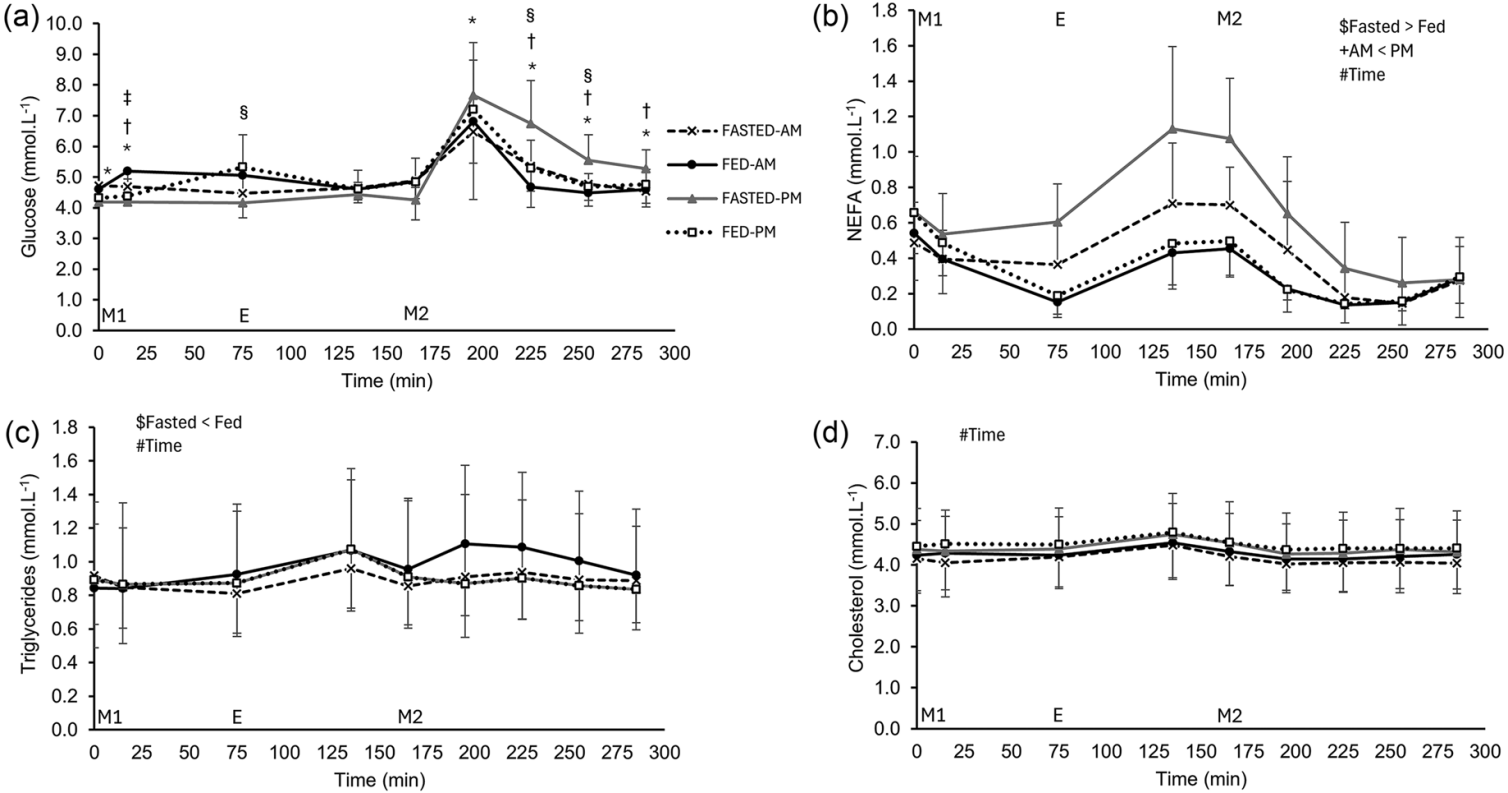
Metabolic marker responses. Serum concentrations over time of glucose (a), NEFA (b), triglycerides (c) and cholesterol (d). Trials involved exercise performed in the fasted state (FASTED) or after consumption of test meal 1 (FED) and in the morning (AM) or evening (PM). Dashed line (crosses), FASTED‐AM; black continuous line (filled circles), FED‐AM; grey continuous line (filled triangles), FASTED‐PM; dotted line (open squares), FED‐PM. ‘M1’ indicates ingestion of test meal 1 during the FED trials or continuation of fasting during the FASTED trials; ‘E’ indicates the start of the exercise period, where participants completed 1 h of cycling at 60% of their ${\dot{V}}_{O_{2}\mathrm{peak}}$; and ‘M2’ indicates ingestion of test meal 2 where 800 g vegetable soup was ingested in all trials. Values represent means ± SD; *n* = 12 participants. *Difference between FAST‐AM and FAST‐PM (*P *< 0.05), †difference between FED‐AM and FAST‐PM (*P *< 0.05), ‡difference between FED‐AM and FED‐PM (*P *< 0.01), §difference between FAST‐PM and FED‐PM (*P *< 0.05). Main effects indicated on figures: $main effect of trial, +main effect of time of day, #main effect of time (*P *< 0.05). NEFA, Non‐esterified fatty acids.

No main effect of trial (*P* = 0.653) or time of day (*P* = 0.276), but a main effect of time (*P *< 0.001) and three‐way interaction effect (*P* = 0.001) was observed for glucose concentrations (Figure 6a). Glucose concentrations were higher in the FASTED‐PM trial in the postprandial period of meal 2 compared to FASTED‐AM at 195 min, 225 min, 255 min and 285 min, FED‐AM at 225 min, 255 min and 285 min and FED‐PM at 225 and 255 min (*P *< 0.05). A main effect of trial (*P *< 0.001), time of day (*P* = 0.003) and time (*P *< 0.001) was observed for NEFA concentrations, although no three‐way interaction effect (*P* = 0.165) resulted. NEFA concentrations were greater in the evenings compared to the mornings (0.48 ± 0.37 vs. 0.36 ± 0.26 mmol L^−1^) and were greater in the fasted compared to the fed trials (0.51 ± 0.38 vs. 0.33 ± 0.23 mmol L^−1^) (Figure 6b). A main effect was found for trial (*P* = 0.009) and time (*P *< 0.001) for triglyceride concentrations, but no time of day (*P* = 0.112) or interaction effect (*P* = 0.167) was observed. Triglyceride concentrations were lower in the fasted trials compared to the fed (0.89 ± 0.28 vs. 1.10 ± 0.45 mmol L^−1^) (Figure 6c). No main effect was found for time of day (*P* = 0.073) or trial (*P* = 0.131), and no interaction effect was present (*P* = 0.135) for cholesterol concentrations. There was a main effect of time, however (*P *< 0.001) (Figure 6d).

**FIGURE 7:**
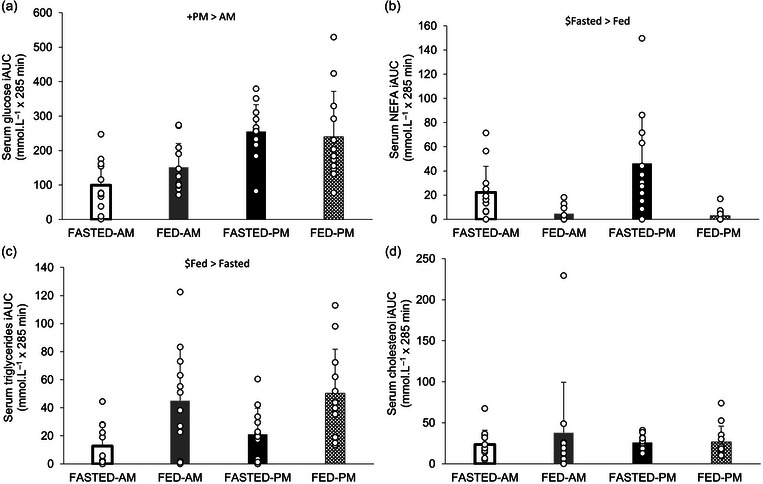
Metabolic marker responses. iAUC serum concentrations of glucose (a), NEFA (b), triglycerides (c) and cholesterol (d). Trials involved exercise performed in the fasted state (FASTED) or after consumption of test meal 1 (FED) and in the morning (AM) or evening (PM). Values represent means ± SD; *n *= 12 participants. The main effects indicated in the figures: $main effect of trial, +main effect of time of day (*P* < 0.05). iAUC, incremental area under the curve; NEFA, non‐esterified fatty acids.

Serum glucose iAUC concentrations showed no main effect of trial (*P* = 0.470) or trial × time of day interaction effect (*P* = 0.111), but a main effect of time of day was observed (*P* = 0.001). Glucose iAUC concentrations were higher in the evening trials in comparison to the morning trials (247 ± 106 vs.125 ± 75 mmol L^−1^ × 285 min) (Figure 7a). NEFA iAUC concentrations showed a main effect of trial (*P* = 0.001) but not time of day (*P* = 0.141) or an interaction effect (*P* = 0.129). NEFA iAUC concentrations were greater in the fasted trials compared to the fed trials (34 ± 35 vs. 4 ± 6 mmol L^−1^ × 285 min) (Figure 7b). A main effect of trial was observed for triglycerides iAUC with fed greater than fasted (47 ± 34 vs. 17 ± 17 mmol L^−1^ × 285 min, *P* = 0.010). No main effect of time of day (*P* = 0.423) or an interaction effect (*P* = 0.799) was observed, however (Figure 7c). No main effect or interaction effect was seen with cholesterol iAUC (trial *P* = 0.408, time of day *P* = 0.685, interaction *P* = 0.478) (Figure 7d).

### 3.4 Appetite

**FIGURE 8:**
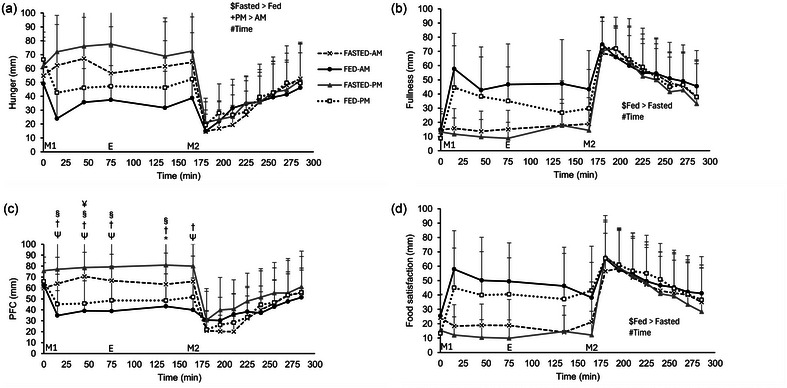
Appetite scores during trials, assessed by 100 mm VAS. (a) Hunger, (b) fullness, (c) PFC, (d) food satisfaction. Trials involved exercise performed in the fasted state (FASTED) or after consumption of test meal 1 (FED) and in the morning (AM) or evening (PM). Dashed line (crosses), FASTED‐AM; black continuous line (filled circles), FED‐AM; grey continuous line (filled triangles), FASTED‐PM; dotted line (open squares), FED‐PM. ‘M1’ indicates ingestion of test meal 1 during the FED trials or continuation of fasting during the FASTED trials; ‘E’ indicates the start of the exercise period, where participants completed 1 h of cycling at 60% of their ${\dot{V}}_{O_{2}\mathrm{peak}}$; and ‘M2’ indicates ingestion of test meal 2 where 800 g vegetable soup was ingested in all trials. Values represent means ± SD; *n *= 12 participants. *Difference between FASTED‐AM and FASTED‐PM (*P *< 0.05), †difference between FED‐AM and FASTED‐PM (*P *< 0.01), §difference between FASTED‐PM and FED‐PM (*P *< 0.05), ¥difference between FASTED‐AM and FED‐PM (*P *< 0.05), Ψdifference between FASTED‐AM and FED‐AM (*P *< 0.05). The main effects indicated in the figures: $main effect of trial, +main effect of time of day (*P *< 0.05), #main effect of time. PFC, prospective food consumption; VAS, visual analogue scale.

A main effect of trial (*P* = 0.002), time of day (*P* = 0.028) and time (*P *< 0.001), but no three‐way interaction effect (*P* = 0.128), was observed for hunger (Figure 8a). Hunger ratings were higher in the evening trials compared to the morning (46 ± 29 vs. 39 ± 28 mm) and higher in the fasted compared to the fed (48 ± 30 vs. 38 ± 27 mm). Meal ingestion decreased hunger which then progressively increased in the postprandial period (*P *< 0.05). There was a main effect of trial (*P* = 0.002) and time (*P* < 0.001) for fullness, although no main effect of time of day (*P* = 0.270) or three‐way interaction effect (*P* = 0.277) (Figure 8b). Fullness ratings were higher in the fed compared to the fasted trials (48 ± 28 vs. 37 ± 29 mm), increasing post‐meal ingestion before progressively decreasing in both postprandial periods (*P *< 0.05). A main effect of trial (*P* = 0.026), time of day (*P* = 0.005) and time (*P* < 0.001) and three‐way interaction effect (*P* = 0.047) was observed for PFC (Figure 8c). PFC was higher in the FASTED‐PM trial compared to both FED trials at 15, 45, 75 and 135 min, whilst in the FASTED‐AM trial PFC was greater than in the FED‐AM trial at 15, 45, 75 and 165 min (*P *< 0.05). Differences in PFC diminished following the ingestion of test meal 2. A main effect of trial (*P* = 0.001), time (*P* < 0.001), but no time of day (*P* = 0.434) or interaction effect (*P* = 0.800) was seen for food satisfaction (Figure 8d). Food satisfaction was greater in the fed trials compared to the fasted (23 ± 27 vs. 18 ± 24 mm) and increased but then progressively decreased in both postprandial periods as with fullness (all *P *< 0.05).

**FIGURE 9:**
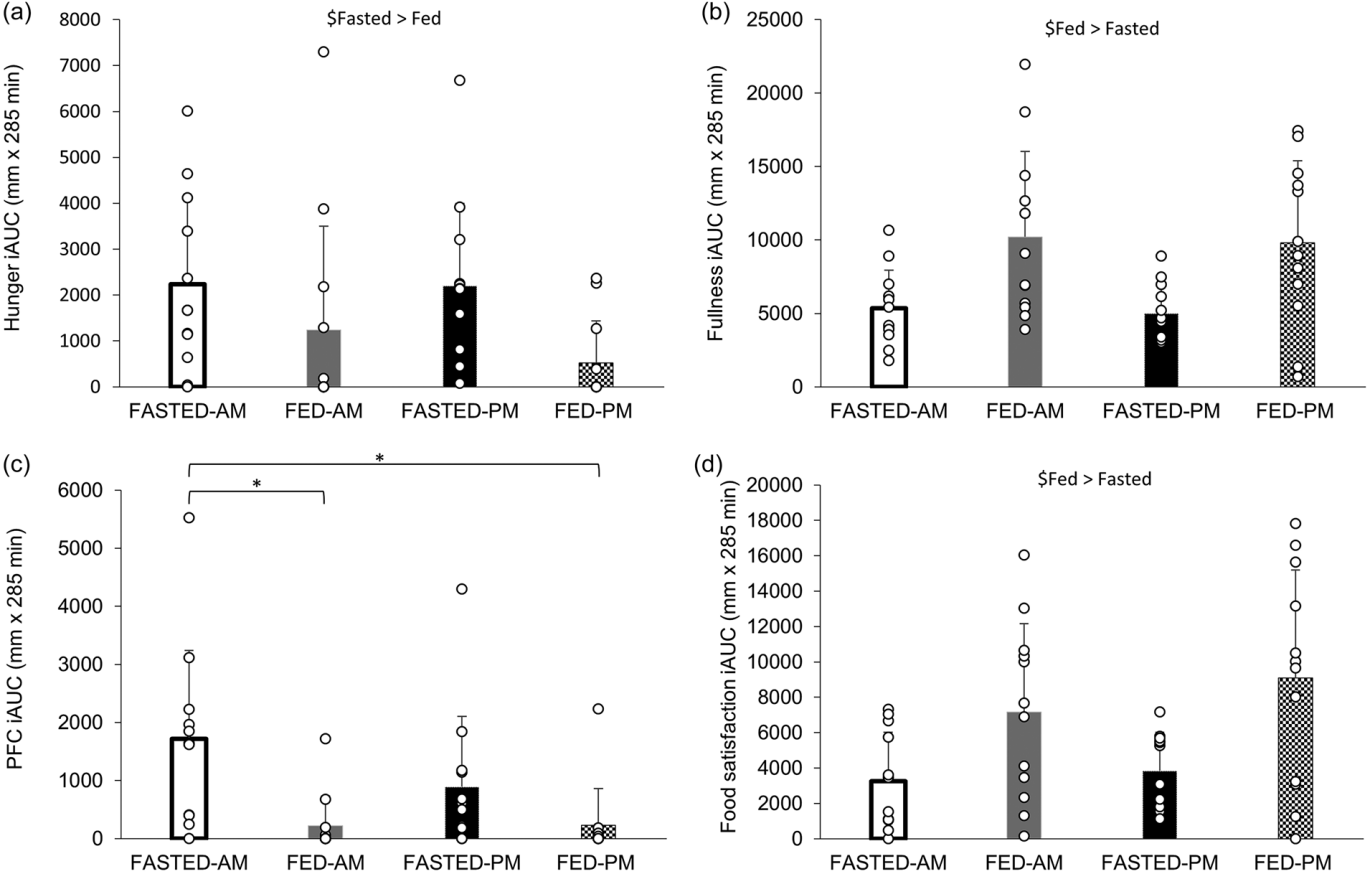
iAUC appetite scores during trials, assessed by 100 mm VAS. (a) Hunger, (b) fullness, (c) PFC, (d) food satisfaction. Trials involved exercise performed in the fasted state (FASTED) or after consumption of test meal 1 (FED) and in the morning (AM) or evening (PM). Values represent means ± SD; *n* = 12 participants. *Difference between trials (*P *< 0.05). Main effects indicated on figures: $main effect of trial (*P *< 0.05). iAUC, incremental area under the curve; PFC, prospective food consumption; VAS, visual analogue scale.

A main trial effect for hunger ratings for iAUC was observed (*P* = 0.002), but no time of day effect (*P* = 0.282) or interaction effect (*P* = 0.317) (Figure 9a). Hunger iAUC ratings were higher in the fasted trials compared to the fed trials (2217 ± 1818 vs. 880 ± 1727 mm × 285 min). A main effect of trial was observed for fullness iAUC (*P* = 0.001; Figure 9b) and food satisfaction iAUC (*P *< 0.001; Figure 9d) with the fed trials greater than fasted (fullness 10,003 ± 5585 vs. 5166 ± 2262 mm × 285 min; food satisfaction 8127 ± 5542 vs. 3548 ± 2451 mm × 285 min). No main effect of time of day or interaction effect was seen, however (fullness, *P* = 0.747 and *P* = 0.982, respectively and food satisfaction, *P* = 0.380 and *P* = 0.564, respectively). A main effect of trial (*P* = 0.001) and time of day (*P* = 0.014) and an interaction effect (*P* = 0.022) was seen for PFC iAUC (Figure 9c). FASTED‐AM was greater than FED‐AM and FED‐PM (1719 ± 1519 vs. 224 ± 510 mm × 285 min, *P* = 0.006; and vs. 226 ± 635 mm × 285 min, *P* = 0.003).

### 3.5 Substrate utilisation

**FIGURE 10:**
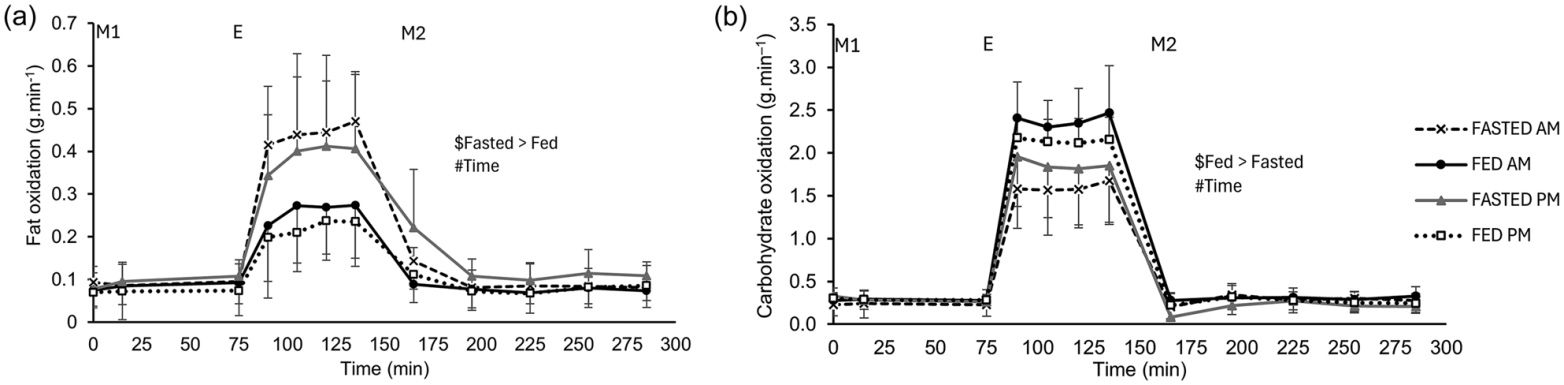
Substrate utilisation. Fat oxidation (a) and CHO oxidation (b) during the trials. Trials involved exercise performed in the fasted state (FASTED) or after consumption of test meal 1 (FED) and in the morning (AM) or evening (PM). Dashed line (crosses), FASTED‐AM; black continuous line (filled circles), FED‐AM; grey continuous line (filled triangles), FASTED‐PM; dotted line (open squares), FED‐PM. ‘M1’ indicates ingestion of test meal 1 during the FED trials or continuation of fasting during the FASTED trials; ‘E’ indicates the start of the exercise period, where participants completed 1 h of cycling at 60% of their ${\dot{V}}_{O_{2}\mathrm{peak}}$; and ‘M2’ indicates ingestion of test meal 2 where 800 g vegetable soup was ingested in all trials. Values represent means ± SD; *n =* 12 participants. Main effects indicated on figures: $main effect of trial, #main effect of time. CHO, carbohydrate.

**FIGURE 11:**
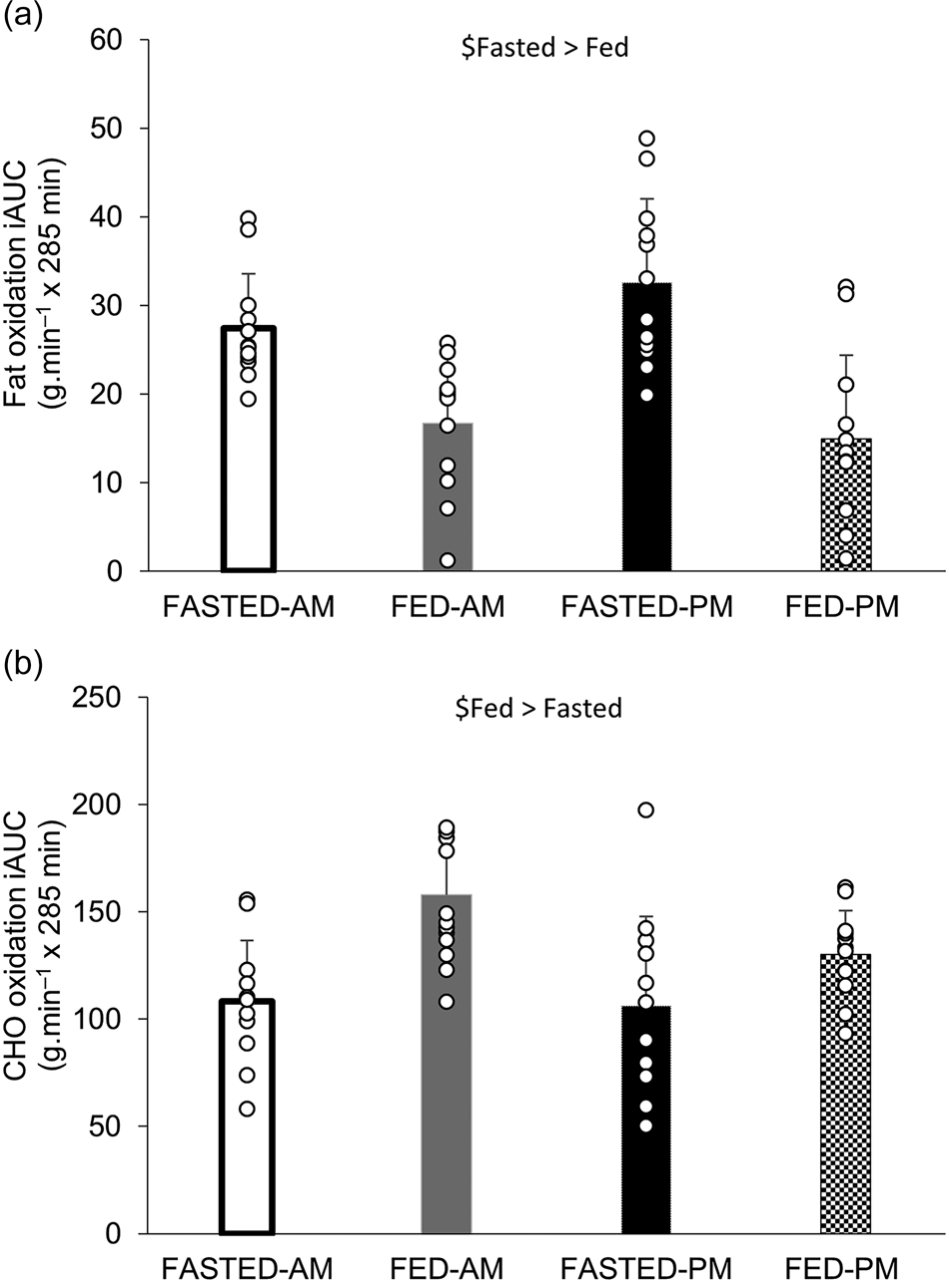
iAUC substrate utilisation. Fat oxidation (a) and CHO oxidation (b) during the trials. Trials involved exercise performed in the fasted state (FASTED) or after consumption of test meal 1 (FED) and in the morning (AM) or evening (PM). Values represent means ± SD; *n =* 12 participants. Main effects indicated in figures: $main effect of trial. CHO, carbohydrate; iAUC, incremental area under the curve.

A main effect of trial (*P *< 0.001) and time (*P *< 0.001), but no time of day (*P* = 0.537) or three‐way interaction effect (*P* = 0.738) was observed for fat oxidation (Figure 10a). Fat oxidation increased with exercise and then decreased with cessation of exercise back to pre‐exercise levels (all *P *< 0.05). Fat oxidation iAUC had no main effect of time of day (*P* = 0.323). There was a main effect of trial with it being greater in the fasted trials compared to the fed trials (30 ± 8 vs. 16 ± 8 g.min^−1^ × 285 min; *P *< 0.001), but there was no interaction effect (*P* = 0.232); Figure 11a). A main effect of trial (*P* < 0.001) and time (*P* < 0.001), but no time of day effect (*P* = 0.785) or three‐way interaction effect (*P* = 0.072), was also observed for CHO oxidation. CHO oxidation increased with exercise and returned to pre‐exercise values following the cessation of exercise (all *P *< 0.05) (Figure 10b). CHO oxidation iAUC had no main effect of time of day (*P* = 0.150). There was a main effect of trial with it being greater in the fed trials compared to the fasted (141 ± 26 vs. 107 ± 35 g.min^−1^ × 285 min, *P* = 0.002), but there was no interaction effect (*P* = 0.191; Figure 11b).

### 3.6 Twenty‐four hour EI

A main effect of time of day where AM was greater than PM (3097 ± 449 vs. 2715 ± 660 kcal, *P* = 0.043) was observed for 24 h EI. However, no main effect of trial (*P* = 0.145) or trial × time of day interaction effect (*P* = 0.096) was observed (Table 2).

**TABLE 2:** Twenty‐four hour post‐trial EI and macronutrient breakdown represented in grams and as a percentage of total EI (*n* = 12 participants).

	FASTED‐AM	FED‐AM	FASTED‐PM	FED‐PM
EI (kcal)	2963 ± 444	3230 ± 431	2701 ± 649	2729 ± 700
Protein (g)	150 ± 22 (20%)	177 ± 29 (22%)	145 ± 24 (21%)	169 ± 25 (23%)
Carbohydrate (g)	314 ± 85 (42%)	332 ± 53 (41%)	262 ± 91 (39%)	275 ± 76 (38%)
Fat (g)	123 ± 35 (38%)	133 ± 45 (37%)	119 ± 43 (40%)	123 ± 38 (39%)

*Note*: Values expressed as means ± SD. Abbreviations: FASTED‐AM, morning fasted; FED‐AM, morning fed; EI, energy intake; FASTED‐PM, evening fasted; FED‐PM, evening fed.

### 3.7 Salivary melatonin and the midpoint of sleep

There was a main effect of time of day (*P* < 0.001), no main effect of trial (*P* = 0.617) and no interaction effect (*P* = 0.901) for salivary melatonin levels. Salivary melatonin concentrations were greater in the mornings compared to the evenings (Table 3). No differences between trials were seen for the midpoint of sleep (*P* = 0.267).

**TABLE 3:** Circadian marker and sleep pattern measurements (*n* = 12 participants).

	FASTED‐AM	FED‐AM	FASTED‐PM	FED‐PM
Melatonin (pg/mL)	15.2 ± 7.2^a^	15.8 ± 9.5^a^	4.9 ± 2.4	5.3 ± 2.2
Midpoint of sleep	02:40 ± 0:07	02:30 ± 0.06	02:32 ± 0.04	02:42 ± 0.03

*Note*: Values expressed as means ± SD. Abbreviations: FASTED‐AM, morning fasted; FED‐AM, morning fed; FASTED‐PM, evening fasted; FED‐PM, evening fed. ^a^Greater than PM trials.

## 4 DISCUSSION

The aim of the study was to investigate if fasted or fed exercise in the morning or evening would result in differences in GER, appetite and metabolic responses. The primary findings were that GER in individuals with overweight was unaffected by fasted exercise or the time of exercise, and overall responses of many of the appetite regulatory hormones, metabolic markers and subjective feelings of appetite were also unaffected by the interaction of metabolic state and time of the day. However, total PP response (iAUC) was greater following evening fed exercise compared to both fasted exercise in the morning and in the evening, whilst other satiety hormone responses of GLP‐1, insulin and PYY showed a main effect of fed being greater than fasted exercise. In addition, glucose concentrations were elevated in the postprandial period of subsequent meal ingestion following fasted exercise in the evening compared to all other conditions. Furthermore, higher ratings of PFC in the FASTED‐PM condition diminished following subsequent meal ingestion. The present findings do not support the hypothesis that individuals with overweight would have differences in GER but partially support the hypothesis that there would be different appetite and metabolic responses to fasted versus non‐fasted exercise, which may be modulated by the time of day.

To the investigators’ knowledge, no studies have assessed the diurnal variation in gastrointestinal function, metabolic markers and appetite regulation in response to fasted versus non‐fasted exercise in individuals with overweight. The lack of diurnal variation of GER observed in this study does not support previous evidence that gastric emptying is delayed in the evening compared to the morning (Goo et al., 1987; Grammaticos et al., 2015) nor that FASTED‐PM exercise results in delayed GER (McIver et al., 2019a). This may be because adiposity outweighs or diminishes any diurnal variation or fasted exercise effect in GER. Previous studies have shown that individuals with overweight display a lessened meal‐induced increase in PYY and GLP‐1 levels (Le Roux et al., 2006; Verdich et al., 2001) and diminished suppression of acylated ghrelin in the postprandial period (Le Roux et al., 2006). The secretion of these satiety hormones and suppression of the hunger hormone in response to a meal plays an important role in the inhibition of GER (Camilleri, 2019). Indeed, the responses of these hormones in the present study did not differ in the postprandial period of the second meal between the morning and evening, although the limitation of low sample size for PYY (*n = *6 participants) should be noted. Alternatively, the semi‐solid nature, the macronutrient composition or the low energy content relative to body mass of the food that was utilised was not a sufficient stimulus for the detection of any differences between conditions in individuals with overweight. The differences in GER in individuals with overweight or obesity compared with lean individuals remain unclear in the extant literature, although the general consensus is that GER is accelerated in individuals with overweight or obesity, with a number of studies supporting this (Bluemel et al., 2017; Clegg & Shafat, 2009; Cardosa‐Junior et al., 2007; Tosetti et al., 1996; Valera Mora et al., 2005). Exploration of diurnal variations on GER within other adiposity cohorts, such as lean individuals and individuals with obesity, is required to confirm whether the lack of differences observed in the current findings is applicable to individuals with overweight only.

The present study found that PP iAUC response was greater in the evening following fed exercise compared to fasted exercise in the morning and evening. This elevated satiety hormone response is in line with the ingestion of two meals during the trial as PP is released during all phases of digestion (Druce et al., 2004), and the diurnal rhythm shown for PP which reaches maximal levels by mid‐afternoon and remains high until the early evening (Johns et al., 2006). The potential implication for this greater response in subsequent appetite regulation is unknown and possibly limited as no differences in appetite ratings or subsequent 24 h EI were seen. Fasted exercise did not elicit a compensation of EI post‐trial. This finding is supportive of studies that have shown that skipping breakfast does not lead to a complete compensation of EI at subsequent meals (Levitsky & Pacanowski, 2013; Chowdhury et al., 2015). The combination of exercise‐induced EE in the present study and greater utilisation of fat as an energy substrate during exercise suggests that fasted exercise may be a beneficial approach to weight loss or management if this acute finding is sustained longer‐term over repeated bouts of fasted exercise. Whether an increase in EI in anticipation of the prolonged fast may occur during free living is also a consideration, though regular fasted exercise training, perhaps on alternate days, may negate this.

Postprandial glucose response was higher following the second meal in the FASTED‐PM trial compared to all other trials. This reduced glycaemic control in the evening may be explained by a combination of the later timing of meal ingestion and the omission of the previous meal. Diurnal variations of glucose concentrations have been shown previously (Stutz et al., 2024; Sato et al., 2011) and ingestion of a meal consumed in the evening evokes a greater postprandial response compared to ingestion earlier in the day (Gibbs et al., 2014; Leung et al., 2019) due to possible decreases in insulin sensitivity that have been found to occur during the day (Carrasco‐Benso et al., 2016) or decreased pancreatic β‐cell function in the evening (Reutrakul & Knutson, 2016). The main effects of time of day and fasted/non‐fasted for GLP‐1 found in the present study, where responses were lower in the evening and with fasted exercise, are congruent with this given GLP‐1's insulinotropic role (Yabe & Seino, 2011). Furthermore, the omission of the previous meal would lead to the absence of the second‐meal phenomenon, which was first discovered by Hamman and Hirschman, 1919, whereby consumption of a prior meal leads to improved glucose tolerance in a subsequent meal. Of note, the glucose responses seen in the present study were not in agreement with the paradoxical second meal phenomenon, where glucose tolerance of a meal is reduced in the postexercise period following prior meal consumption (Gonzalez, 2014). With the paradoxical second meal phenomenon, one would have expected the two fed exercise trials to have had greater glucose responses than the two fasted trials. One potential reason may be the timing of the second meal ingestion following 30 min of recovery as opposed to more immediately after the cessation of exercise.

Although there was a greater rating of PFC in the FASTED‐PM condition, the differences diminished following subsequent meal ingestion post‐exercise. There were also no differences in hunger and fullness in this same period. This is likely explained by the minimal differences in appetite regulatory hormone responses also observed in this study. These minimal or lack of differences may be due to the participants being individuals with overweight, as having higher body fat is linked with disrupted and blunted satiety signalling in the postprandial phase (Lean & Malkova, 2016; Flint et al., 2007). Hormonal appetite responses in individuals with overweight/obesity following an acute bout of exercise have been found to show subtle differences with greater exercise‐induced suppression of desacylated ghrelin and greater total GLP‐1 response but lower total PYY response compared to lean individuals (Douglas et al., 2017). Whether there are differences in acute appetite hormone responses to fasted exercise at different times of the day within lean individuals and individuals with obesity is unknown.

There are some limitations to the current study that need acknowledging; first, only male participants were recruited within the study, and participants were self‐reported as recreationally active, partaking in more than the recommended 150 min of moderate‐intensity physical activity per week. It is unknown how female participants and those who are more sedentary may respond. The present study also had a relatively short postprandial period of 2 h and a semi‐solid meal was utilised. As individuals with overweight or obesity have a delayed postprandial onset of satiation and reduced fullness (Bluemel et al., 2017; Delgado‐Aros et al., 2004), a longer postprandial period, and ingestion of a solid meal may be needed to discover any effects of fasted exercise on GER and appetite responses. In addition, although the study was designed to investigate the effect of a combination of intermittent fasting and exercise by omitting/skipping the ingestion of a meal to evoke a fasted state for exercise, it can be argued that it would have been difficult to attribute any differences seen within the trial duration to exercising in the fasted state per se rather than the reduced EI in comparison to the fed trials. An alternative design and aim would be to investigate energy‐matched fed trials by providing test meal 1 after the performance of the fasted exercise. However, this study measured 24‐h EI after the trial to account for the omission of test meal 1 EI during the fasted exercise conditions to explore if any compensatory EI occurred as a result, which was not found.

In conclusion, GER does not follow a diurnal variation and was not affected by fasted exercise in individuals with overweight. Fasted exercise also did not provoke compensatory effects on appetite regulatory responses or subjective feelings of hunger during the trial or 24 h post‐trial EI in individuals with overweight. Thus, the performance of moderate‐intensity exercise in the fasted state may be an effective approach for weight management. However, fasted exercise in the evening may lead to poorer glucose control in individualswith overweight. Further work is required to explore the longer‐term effects of fasted versus non‐fasted exercise on appetite, weight control and metabolic regulation in individuals with overweight as well as other population groups of varying body composition.

## AUTHOR CONTRIBUTIONS

This study was performed in the Physiology Research Laboratories in the Faculty of Science and Engineering at Manchester Metropolitan University. Victoria Joan McIver and Adora Mo Wah Yau conceived and designed the experiments; Victoria Joan McIver, Adora Mo Wah Yau, Gethin Hywel Evans, and Lewis Robert Mattin acquired the data; Victoria Joan McIver and Adora Mo Wah Yau analysed and interpreted the data; Victoria Joan McIver drafted the manuscript with revisions from Adora Mo Wah Yau and Gethin Hywel Evans. All authors have read and approved the final version of this manuscript and agree to be accountable for all aspects of the work in ensuring that questions related to the accuracy or integrity of any part of the work are appropriately investigated and resolved. All persons designated as authors qualify for authorship, and all those who qualify for authorship are listed.

## CONFLICT OF INTEREST

None declared.

## Data Availability

Data will be shared upon reasonable request. Ethical approval for this study was granted on the basis that data will not be made available on an open‐access repository.
